# Complex Assessment of the Incidence and Risk Factors of Delirium in a Large Cohort of Cardiac Surgery Patients: A Single-Center 6-Year Experience

**DOI:** 10.1155/2013/835850

**Published:** 2013-12-22

**Authors:** Łukasz J. Krzych, Maciej T. Wybraniec, Irena Krupka-Matuszczyk, Michał Skrzypek, Anna Bolkowska, Mirosław Wilczyński, Andrzej A. Bochenek

**Affiliations:** ^1^Department of Cardiac Surgery, Upper Silesia Medical Center, Medical University of Silesia, 47 Ziołowa Street, 40-635 Katowice, Poland; ^2^Department of Anesthesiology and Intensive Care, Upper Silesia Medical Center, 40-635 Katowice, Poland; ^3^1st Department of Cardiology, Upper Silesia Medical Center, Medical University of Silesia, 40-635 Katowice, Poland; ^4^Department of Psychiatry and Psychotherapy, Upper Silesia Medical Center, Medical University of Silesia, 40-635 Katowice, Poland; ^5^Department of Biostatistics, Chair of Public Health, Medical University of Silesia, 41-902 Bytom, Poland

## Abstract

*Background*. Previous reports provided inconsistent data on the occurrence of postoperative delirium and emphasized its considerable impact on outcome. This study sought to evaluate the incidence and predictors of delirium, together with its relation to cerebral ischemia in a large cohort of cardiac surgery patients in a tertiary high-volume center. *Methods and Results*. Consecutive patients (*n* = 8792) were prospectively enrolled from 2003 to 2008. Exclusion criteria were history of psychiatric disorders, use of psychoactive drugs, alcohol abuse, and data incompleteness. Finally, 5781 patients were analyzed in terms of 100 perioperative patient-specific and treatment variables. The incidence of postoperative delirium (DSM IV criteria) was 4.1% and it coexisted with cerebral ischemia in 1.1% of patients. In bivariate analysis, 49 variables were significantly linked to postoperative delirium. Multivariate analysis confirmed that delirium was independently associated with postoperative stroke (logistic odds ratio (logOR) = 2.862, *P* = 0.004), any blood transfusions (logOR = 4.178, *P* < 0.0001), age > 65 years (logOR = 2.417, *P* = 0.002), carotid artery stenosis (logOR = 2.15, *P* = 0.01), urgent/emergent surgery (logOR = 1.982, *P* = 0.02), fasting glucose level, intraoperative oxygen partial pressure fluctuations, and hematocrit. Area under ROC curve for the model was 0.8933. *Conclusions*. Early identification of nonpsychiatric perioperative determinants of delirium facilitates its diagnosis and might help develop preventive strategies to improve long-term outcome after cardiac surgery procedures.

## 1. Introduction

Despite the undeniable progress in surgical techniques and perioperative care of cardiac patients, composite rate of mortality and postoperative morbidity still remains considerable, which is vastly attributable to neuropsychiatric complications [1, 2]. According to American College of Cardiology/American Heart Association (ACC/AHA) classification, postoperative neuropsychological disorders can be categorized into major permanent or transient focal neurological deficits (type 1) or cognitive impairment and immediate decline in intellectual function (type 2) [3]. Delirium as the most common example of neurologic injury type 2 represents an acute confusional state affecting from 2 to even 70% of patients undergoing cardiac surgery procedures [4–8]. Numerous studies denoted its association with prolonged hospitalization time in the intensive care unit (ICU) and increased total in-hospital stay [4, 7, 9–13], extended time of mechanical ventilation [13], increased risk of infection (including pneumonia [11–13] and sternal wound infection [4]), decreased health-related quality of life [9], and, most importantly, higher long-term mortality [6]. All of them are associated with greater financial burden on the healthcare system.

Since the available management is purely symptomatic (neuroleptic and sedative agents) [14] and auxiliary [15–17], preventive approach to modifiable nonpsychiatric perioperative risk factors of delirium merits substantial consideration. Remarkable variation in the prevalence of delirium requires further investigation. Finally, recently published data on potential predictors of delirium are rather scarce and not supported by large cohort studies [4–7, 9, 11, 18–21]. Complex assessment of delirium helps create a universal and adaptable scoring system to facilitate decision-making in everyday clinical practice in patients with delirium [22]. Thus we intended to investigate into the incidence and nonpsychiatric determinants of delirium in a large cohort of cardiac surgery patients.

## 2. Methods

### 2.1. Patients

**Figure 1:**
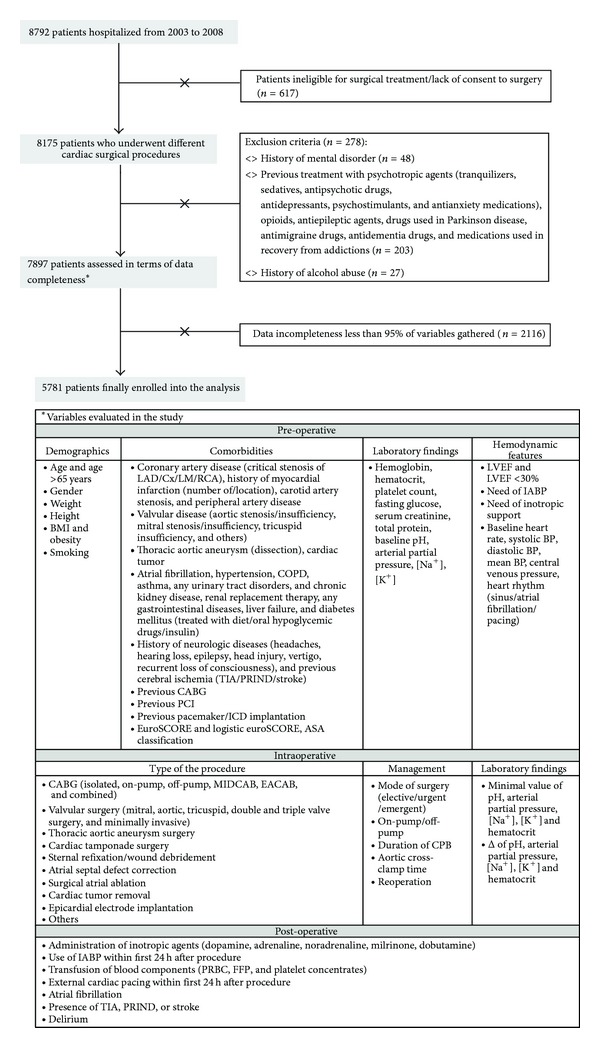
Study design and patients' flow chart. ASA: American Society of Anesthesiologists classification, BMI: body mass index, BP: blood pressure, CABG: coronary artery bypass grafting, COPD: chronic obstructive pulmonary disease, ICD: implantable cardioverter—defibrillator, LAD: left anterior descending branch, Cx: circumflex branch, IABP: intraaortic balloon pump, LM: left main coronary artery, LVEF: left ventricle ejection fracture, PCI: percutaneous coronary intervention, RCA: right coronary artery, and TIA: transient ischemic attack.

The study was performed in a tertiary high-volume cardiac surgery center. Consecutive patients were prospectively enrolled between January 2003 and December 2008. A flow chart of subjects is delineated in Figure 1. The exclusion criteria were as follows: any psychiatric disorder in the past, preoperative use of psychoactive drugs, and history of alcohol abuse. A total of over 100 pre-, intra- and postoperative nonpsychiatric variables were assessed (Figure 1). Only the subjects to at least 95% of known parameters were incorporated into the database. Finally, 5781 patients were included (1750, 30.3% women and 4031, 69.7% men). The study complied with the Declaration of Helsinki and was approved by the local Ethics Committee. On admission all patients gave their written consent to personal medical data processing for the purpose of this study.

### 2.2. Methods and Definitions

Delirium was diagnosed postoperatively by the attending physician (cardiac surgeon or intensive care specialist) according to the criteria of Diagnostic and Statistical Manual of Mental Disorders IV edition (DSM-IV) [23], which are regarded as the most inclusive definition of delirium [24]. Afterwards, every case was confirmed by a consulting psychiatrist. Any suspicion of cerebral ischemia was followed by a diligent neurological examination by a consulting neurologist. Should it be for unclear diagnosis or differentiation between hemorrhagic or ischemic form of stroke. Computed tomography was conducted. Cerebral ischemia was defined as transient ischemic attack (TIA) or stroke. TIA was diagnosed when symptoms of cerebral ischemia reversed within 24 h and stroke when symptoms persisted longer than 24 h after the onset of neurological deficits. The presence of concurrent comorbidities (e.g., diabetes, arterial hypertension, and atrial fibrillation) was ascertained through diligent review of referral letters and discharge summaries provided by patients or diagnosed primarily during current hospitalization. The presence, extent and topography of coronary artery disease were determined by means of preoperative coronary angiography. Left ventricular ejection fraction and the presence of valvular heart disease were verified with 2D echocardiography. Heart function was classified depending on ejection fraction into good (>50%), moderate (30–50%), and poor (<30%). The extent of carotid artery stenosis was verified preoperatively with Doppler ultrasonography. Additive EuroSCORE (in points) and logistic EuroSCORE (in %) were calculated. Perioperative risk was categorized into: low (0–2 points), moderate (3–5 points) or high (6+ points) [25]. Physical status by the American Society of Anesthesiologists (ASA) classification was assessed. Laboratory tests were performed within 24 h before and after the procedure. Arterial blood gas analysis was performed in samples collected from radial artery in the operating room. All measurements were consistent with ISO 9001:2008. The hemodynamic variables were collected on the day of surgery before induction of anesthesia (continuous electrocardiographic monitoring, invasive arterial blood pressure monitoring, central venous pressure measured through jugular or subclavian access). To investigate the importance of intraoperative variations/fluctuations (Δ = maximal *minus* minimal value) of arterial blood gas parameters (including oxygen partial pressure, partial pressure of CO_2_, pH, [K^+^], [Na^+^], hemoglobin concentration and hematocrit) during the surgery under general anesthesia (with 100% oxygen supply) we categorized these variables on the basis of their quartile distribution, taking <ΔQ1 as the cutoff for extremely low, ΔQ1–3 as a reference interval of no variation and >ΔQ3 as the cutoff for extremely high variations.

### 2.3. Anesthetic and Surgical Management

All patients were anesthetized in coherence with the unified protocol involving oral premedication with midazolam (7.5 mg–15 mg) and induction with intravenous etomidate (0.15 mg/kg) or propofol (1.0–2.5 mg/kg) and fentanil (7.0–10.0 *μ*g/kg) and the use of non-depolarizing muscle relaxant (pancuronium 1.0-2.0 mg/kg or cisatracurium 0.15–0.2 mg/kg). The anesthesia was sustained with the technique of total intravenous anesthesia (TIVA; midazolam 1.5–2.0 *μ*g/kg/min, propofol 0.05–0.25 mg/kg/min, or fentanil 0.15 *μ*g/kg/min) or with combined intravenous and inhalational anesthesia (sevoflurane, isoflurane or desflurane). Muscle relaxation was sustained with the repeated doses of muscle relaxant. Intubation was achieved using 6.5–9 mm single- or double-lumen endotracheal tube (for one lung ventilation). We used pressure-control or volume-control mechanical ventilation with 100% oxygen, simultaneously monitoring respiratory rate and volume, airway pressure, end-tidal-CO_2_ and blood oxygen saturation. Patients were hemodynamically monitored in a continuous fashion (electrocardiography: II and V5 lead, invasive intra-arterial blood pressure monitoring, and and central venous pressure measurement with the use of triple-lumen catheter introduced via Seldinger technique). Body temperature was measured using thermistor-based esophageal temperature probe. Foley catheter was inserted and urine output was continuously assessed. Antibiotic prophylaxis of 1st generation cephalosporin was administered to every operated patient for at least 72 h after induction. Intravenous fluids were supplemented at the rate of 80–150 mL/h depending on hydration status (preferably crystalloid solutions).

Surgical management varied depending on the type of procedure and surgeon's preference. Median sternotomy was the predominant approach with the exception of minimally invasive surgeries which involved lateral thoracotomy. On-pump cardiac surgery involved administration of full dose low molecular weight heparin (3 mg/kg) to achieve a target-activated clotting time (ACT) of ≥480 seconds before commencement of cardiopulmonary bypass (CPB), along with the insertion of cannula into the ascending aorta and right atrium. In the off-pump group, heparin (1 mg/kg) was administered before the start of the first anastomosis to achieve an ACT of 250 to 350 seconds. High-potassium (20–30 mmol/l) cardioplegic solution (4 : 1) was reversely injected into the coronary arteries (initially 800–1000 mL, subsequently repeated doses of 200–300 mL). Standard circuit tubing set was used, which included a roller pump and a membrane oxygenator with the flow rate throughout bypass of 2.0–3.0 l/m^2^/min and pressure of 60–70 mmHg. The majority of surgeries were conducted in moderate hypothermia (32°C), except for the aortic dissection surgery requiring deep hypothermia. Rewarming rate was 0.5–1.0°C/5 min. On completion of all anastomoses, protamine was given to reverse the effect of heparin and return the ACT to preoperative levels.

### 2.4. Statistical Analysis

Statistical analysis was performed using SAS 9.2 (SAS Institute, Gary, NC, USA) software. Continuous variables are expressed as mean and standard deviation (normally distributed) or as median and interquartile range (IQR) (nonnormally distributed). The type of distribution was verified using Shapiro-Wilk test. Qualitative variables are expressed as crude values and percent. Between-group differences for normally distributed quantitative variables were assessed using Student *t*-test or analysis of variance, and Mann-Whitney *U*-test or Kruskal-Wallis test were used for those nonnormally distributed. As far as qualitative variables are concerned, Mantel-Haenszel chi-square or Fisher's exact test was applied. In bivariate analysis, delirium (with/without cerebral ischemia) was defined as a dependent variable, whereas independent variables were designated from amongst perioperative parameters. Odds ratios (OR) with 95% confidence intervals (CI) were calculated. Variables with a “*P*” value < 0.1 were consecutively subjected to a multivariate stepwise logistic regression model. Logistic ORs with 95% CIs were subsequently estimated. After the assessment of area under the receiver operator curve, the goodness of fit for logistic regression model was verified with Hosmer-Lemeshow test. A “*P*” value < 0.05 was considered significant.

## 3. Results

The characteristics of the study population are shown in Table 1. Median additive EuroSCORE was 5 points (IQR 3;8) and mean logistic EuroSCORE was 4.78 ± 4.53%. Low risk by EuroSCORE was found in 1436 (24.8%), medium is in 2506 (43.4%) and high risk in 1839 (31.8%) of the subjects. Most of the patients had ASA class III or IV (5600 persons, 96.9%). Surgical data is summarized in Table 2. Median cardiopulmonary bypass time was 77 min (IQR 59;100) and median cross-aortic clamp time was 46 min (IQR 35;63).

Delirium developed postoperatively in 236 subjects (4.1%; 95% CI: 3.57–4.59) and cerebral ischemia alone was present in 148 patients (2.6%; 95% CI: 2.15–2.97). Coexistence of delirium and cerebral ischemia occurred in 65 patients (1.1%; 95%: 0.85–1.39). Median time of delirium was 3 days (IQR 2;5) for those without and 6 days (IQR 2;9) for the patients with cerebral ischemia (*P* < 0.001). Compared to non-psychotic population, delirium significantly prolonged (*P* < 0.001) the time of ICU stay by 3.5 days (IQR 2;5) for subjects without and by 6.5 days (IQR 2;9) for those with cerebral ischemia. Total inhospital stay was also significantly longer (*P* < 0.001) by 5 days (IQR 4;6) for those without and by 9 days (IQR 5;12) for those with cerebral ischemia.

In bivariate analysis it was found that 49 variables had a significant (*P* < 0.1) association with postoperative delirium regardless of cerebral ischemia (Tables 3 and 4). Postoperative cerebral ischemia which approximately 25 times increased the risk of delirium (OR = 25.01, *P* < 0.0001), was the most powerful single determinant of delirium. Conversely, past medical history of cerebral ischemia failed to show any association with perioperative delirium. Among other determinants of neuropsychological complications, a noteworthy association was found for packed red blood cells transfusion (OR = 5.07, *P* < 0.0001), age older than 65 years (OR = 2.58, *P* < 0.0001), urgent mode of surgery (OR = 2.54, *P* < 0.0001), high perioperative risk calculated with EuroSCORE (OR = 2.32, *P* < 0.0001) and a history of chronic obstructive pulmonary disease (OR = 2.06, *P* = 0.005). Although we reported only 16 cases of former CABG, the need for recurrent surgical revascularization strongly correlated with the risk of neuropsychiatric complications (OR = 5.39, *P* = 0.003).

**Figure 2:**
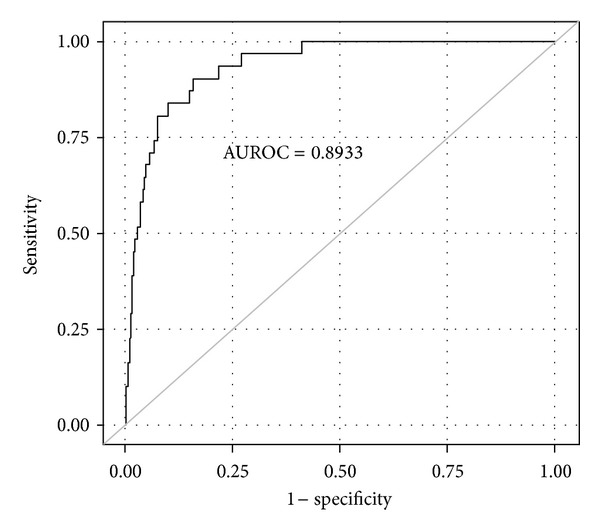
Receiver operator curve (ROC) describing diagnostic accuracy of statistical model in the prediction of delirium. AUROC: area under receiver operator characteristic curve.

Subsequent stepwise logistic regression analysis finally revealed 9 nonpsychiatric variables as independent predictors of psychosis (Table 5), including postoperative cerebral ischemia, any perioperative blood transfusions, older age (>65 years), carotid artery stenosis, nonelective surgery, hypertension, fasting glucose level, high variations of partial oxygen pressure during the procedure, and high variations of hematocrit. The regression equation was of excellent diagnostic accuracy (AUROC = 0.8933) with a Hosmer-Lemeshow test “*P*” value of 0.2 (Figure 2).

Additional analysis in patients who underwent CABG and/or valve procedure revealed similar findings (Table 6); however, only 7 predictors of delirium were placed in a final regression model, including postoperative cerebral ischemia, obstructive pulmonary disease, any perioperative blood transfusions, older age (>65 years), carotid artery stenosis, high variations of partial oxygen pressure during the procedure and any liver disease in the anamnesis. The regression equation was also of excellent diagnostic accuracy (AUROC = 0.9081) with a Hosmer-Lemeshow test “*P*” value of 0.02.

## 4. Discussion

The overall prevalence of postoperative delirium was 4.1%, whereas cerebral ischemia concerned 2.6% of the subjects submitted to cardiac surgery over a 6-year period. In the study of Bucerius et al. [5], namely, the largest cohort of subjects so far, the prevalence of delirium was slightly higher and reached 8.4%. However, the above-cited study utilized DSM-III criteria and did not take into account the occurrence of cerebral ischemia. Different rates of postoperative delirium can be explained by the abundance of diagnostic criteria, which vary in terms of sensitivity [24].

Although postoperative cerebral ischemia affected only 2.6% patients, our analysis indicated stroke as the most reliable predictor of delirium. In line with present study, Manji et al. documented that postoperative occurrence of seizures (also related with cerebral hypoperfusion) corresponds with an increased rate of neuropsychological derangements, defined as delirium and/or stroke, as opposed to non-epileptic individuals (19.6% versus 3.2%, *P* < 0.001) [26]. The reduction of cerebral blood flow is often caused by carotid stenosis, which was a significant predictor of delirium and was the only parameter of atherosclerosis which remained an independent determinant in multivariate analysis. Advanced age, beyond doubt, significantly increases risk of delirium but is also in direct relation with numerous comorbidities (e.g., atrial fibrillation, chronic obstructive lung disease, diabetes, heart failure, renal failure, etc.) which were found to be the predictors of delirium in previous studies [5, 7, 11, 18, 20, 21]. The need for blood transfusion was associated with increased risk of delirium. This may reflect either patient's critical general condition with underlying initial indication for transfusion (e.g., severe bleeding, hemorrhagic diathesis, excessive hemodilution, hemolytic anemia) which causes hypotension, decreased brain perfusion and regional/general hypoxia or the influence of transfusion itself (e.g., immunization, volume overload). Excessive intraoperative fluctuations of arterial blood oxygen saturation and changes of hematocrit were significantly associated with postoperative psychiatric complications. These variables are bound with cerebral hypoxia. It seems advisable to assure adequate and well-balanced anesthetic intraoperative management with cautious preoxygenation. It is reasonable to avoid excessive hemodilution with restrictive fluid resuscitation. Last but not least, the relationship between increased initial fasting glucose level and the onset of delirium is an interesting finding, reflecting the metabolic aspect of delirium's pathogenesis. We also found some protective effect of hypertension (regardless of the coexistence of stroke) that is in contrary to recently published data from INTERSTROKE study, which documented that hypertension is related with a 4-fold higher risk of cerebral complications [27] and an acknowledged study by Roach et al., in which high blood pressure appeared to be the strongest predictor of neuropsychiatric complications after cardiac surgery [28]. The rationale of this observation is vague but we may assume that it is connected with proper control of blood pressure before the surgery, protective effect of pharmacological agents (e.g., *β*-blockers, angiotensin-converting enzyme inhibitors), as well as decreased variations of blood pressure during the cardiopulmonary bypass [29].

### 4.1. Study Limitations

The study has several shortcomings, which may limit application of its results to all populations. First, it is based on the experience of a single medical center. On the one hand, it insures data and procedure consistency, but on the other hand, application of our results to other patient populations is limited. Second, although we enrolled consecutive patients, some of them were excluded due to data incompleteness. Consequently, only about 2/3 of patients were included, which may potentially be the source of selection bias. More to the point, this prospective observational study was conducted between 2003 and 2008 and may not address some advances of perioperative management in the last five years. Third, as the study population is relatively old, the impact of dementia is of great importance, and in some cases clinical differentiation between delirium and cognitive disorders was difficult or even impossible. Moreover, procedure-related depression and anxiety were not investigated. Fourth, it could result in underestimation of delirium prevalence that the diagnosis of delirium was not based upon uniform bed-site algorithms, such as Confusion Assessment Method (CAM), Delirium Observation Screening (DOS), or Delirium Rating Scale (DRS). However, the proximity of tertiary psychiatric center and a meticulous assessment during a routine checkup at least twice a day should have provided acceptable sensitivity of delirium screening. We realize that it requires additional psychological tests and a long-term observation so we decided to exclude patients with any psychiatric disorders in anamnesis and to assess nonpsychiatric variables only. Fifth, the occurrence of cerebral ischemia could be underestimated (silent ischemia with no deficits) because only those subjects with evident neurologic disorders underwent computed tomography examination. Although numerous medications may increase likelihood of delirium, the authors were unable to evaluate their impact on the results because of the heterogeneity of pharmacological treatment. For the same reason this study did not aim at evaluating the significance of perioperative pharmacological neuroprotection, which has been recently brought up by several investigators [30–32]. Finally, the analysis did not cover the presence of aortic atherosclerosis, which is widely recognized as a one of the most crucial risk factors of postoperative stroke [33]. This fact derives from data incompleteness as echocardiography (both transthoracic and transesophageal) is characterized by inadequate sensitivity and computed tomography of aorta was rarely ordered [34]. According to ACCF/AHA guidelines [35], epiaortic ultrasonography is the imaging technique of choice in the detection of aortic atherosclerosis, yet it was not routinely performed within the specified time frame of the study in our center.

## 5. Conclusions

Delirium belongs to frequent psychiatric complications in cardiac surgery setting. Most of nonpsychiatric predictors of delirium are in direct relation with decreased cerebral perfusion and oxygen supply. Improper perioperative anesthetic management and the complications ensuing from the urgency of surgery are of great clinical significance. Early identification of the determinants of delirium may facilitate its diagnosis. It is advisable to utilize the abovementioned risk factors in the bed-site algorithms of perioperative risk assessment to improve the outcome of cardiac surgery patients with psychosis.

## Figures and Tables

**Table 1:** Characteristics of the study group.

*Demographic features *	
Male gender (*n*, %)	4031 (69.7%)
Age (years)	62.8 ± 9.9
Body mass index (kg/m²)	27.5 (24.9; 30)

*Clinical features *	
Hypertension (*n*, %)	4101 (71%)
Diabetes (*n*, %)	1447 (25%)
Atrial fibrillation (*n*, %)	673 (11.6%)
Previous myocardial infarction (*n*, %)	2340 (40.5%)
Previous percutaneous coronary intervention (*n*, %)	821 (14.2%)
Previous coronary artery bypass grafting (*n*, %)	16 (0.2%)
Carotid artery stenosis (*n*, %)	974 (7.9%)
Peripheral artery disease (*n*, %)	810 (14%)
Pacemaker/implantable cardioverter-defibrillator (*n*, %)	50 (0.86%)
Chronic kidney disease (*n*, %)	147 (2.5%)
Renal replacement therapy (*n*, %)	38 (0.6%)
Chronic obstructive pulmonary disease (*n*, %)	219 (3.8%)
Smoking habit (*n*, %)	1738 (30%)
Neurologic diseases (*n*, %)	851 (14.7%)
Stroke/transient ischemic attack (*n*, %)	303 (5.2%)

*Coronary angiography findings *	
Coronary artery disease (*n*, %)	4776 (82.6%)
(1) Vessel disease	2694 (46.6%)
(2) Vessel disease	1937 (33.5%)
(3) Vessel disease	1150 (19.9%)
Left main artery stenosis (*n*, %)	741 (12.8%)

*Echocardiographic features *	
Left ventricular ejection fraction (%)	50.9 ± 10.5
Good (*n*, %)	2858 (49.4%)
Moderate (*n*, %)	1215 (21.0%)
Poor (*n*, %)	1708 (29.6%)
Mitral valve insufficiency (*n*, %)	1020 (17.6%)
Mitral valve stenosis (*n*, %)	247 (4.3%)
Tricuspid valve insufficiency (*n*, %)	297 (5.1%)
Aortic valve insufficiency (*n*, %)	603 (10.4%)
Aortic valve stenosis (*n*, %)	573 (9.9%)

*Laboratory findings *	
Hemoglobin (g/dL)	13.7 ± 1.6
Hematocrit (%)	40.4 ± 4.6
Fasting glucose level (mg/dL)	103 (91; 128)
Oxygen partial pressure (mmHg)	82.3 ± 14.7

*Major hemodynamic features *	
Heart rate (1/min)	71.1 ± 14.6
Systolic blood pressure (mmHg)	133.7 ± 27.0
Diastolic blood pressure (mmHg)	69.3 ± 13.1

**Table 2:** Cardiac surgery procedures.

Type of procedure	Value
*Coronary artery bypass grafting *	4680 (81%)
Isolated	4076 (70.5%)
On-pump	3709 (64.2%)
Off-pump	368 (6.4%)
Combined with valve/septal defect/aneurysm surgery	604 (10.5%)
*Valve surgery *	1290 (22.3%)
Mitral valve surgery	610 (10.6%)
Aortic valve surgery	774 (13.4%)
Double valve surgery	200 (3.5%)
Triple valve surgery	16 (0.3%)
*Other procedures *	513 (8.8%)

**Table 3:** Significant predictors of delirium in a bivariate analysis-qualitative variables.

Variable	OR	95% CI	“*P*”
Patient-related conditions			
Age > 65 years	2.58	1.94–3.43	<0.0001
Coronary artery disease	1.66	1.09–2.55	0.01
Left anterior descending artery stenosis	1.76	1.14–2.72	0.007
Circumflex artery stenosis	1.56	1.07–2.29	0.01
Right coronary artery stenosis	1.73	1.18–2.54	0.003
Previous myocardial infarction	1.39	1.05–1.85	0.01
Carotid stenosis	1.69	1.23–2.31	0.0006
Peripheral artery disease	1.69	1.21–2.36	0.001
Previous percutaneous coronary intervention	2.52	1.64–3.84	<0.0001
Previous coronary artery bypass grafting	5.39	1.21–20.34	0.003
Mitral valve insufficiency	1.41	1.02–1.94	0.03
Hypertension	0.47	0.36–0.61	<0.001
Chronic obstructive chronic disease	2.06	1.19–3.53	0.005
Liver failure	0.23	0.09–0.53	<0.0001
Ulcerous disease	0.70	0.46–1.05	0.07
Diabetes	1.66	1.25–2.21	<0.0001
Any neurological disorders in anamnesis	1.77	1.28–2.44	<0.0001
Urgent/emergent surgery	2.54	1.92–3.34	<0.0001
EuroSCORE ≥ 6 points	2.32	1.63–3.31	<0.0001

Procedure-related conditions			
Postoperative cerebral ischemia	25.01	17.2–31.36	<0.0001
Coronary artery bypass grafting	1.42	0.98–2.07	0.06
On-pump surgery	1.72	1.02–2.93	0.03
Need of inotropic support (postoperatively)	0.43	0.32–0.58	<0.0001
Dopamine use (postoperatively)	0.44	0.33–0.58	<0.0001
Intra-aortic balloon pump (perioperatively)	1.60	1.04–2.44	0.02
Any blood transfusions (perioperatively)	4.65	3.53–6.13	<0.0001
Packed red blood transfusion	5.07	3.85–6.69	<0.0001
Fresh frozen plasma transfusion	2.69	1.96–3.69	<0.0001
Platelet transfusion	4.19	2.72–6.43	<0.0001

CI: confidence interval, OR: odds ratio.

**Table 4:** Significant predictors of delirium in a bivariate analysis-quantitative variables.

Variable	Delirium (+)	Delirium (−)	“*P*”
Patient-related conditions			
Age (years)	69 (62; 74)	63 (56; 70)	<0.001
EuroSCORE (points)	7 (5; 9)	5 (3; 8)	<0.001
Logistic EuroSCORE (%)	5.68 (3.76; 9.91)	3.29 (2.1; 5.7)	<0.001
Hemoglobin (g/dL)	13.6 (12.2; 14.7)	13.9 (12.9; 14.8)	0.009
Hematocrit (%)	39.8 (36.1; 42.6)	40.9 (37.9; 45.5)	<0.001
Platelet count (1000/mm³)	182 (148; 217)	190 (157; 229)	0.005
Fasting glucose level (mg/dL)	120.5 (96; 161.5)	103 (91; 126)	<0.001
Total protein (g/dL)	6.89 (6.3; 7.22)	7.08 (6.6; 7.4)	<0.001
Baseline K^+^ (mmol/L)	3.91 (3.7; 4.16)	3.82 (3.64; 4.06)	0.002
Baseline Na^+^ (mmol/L)	139.7 (138; 141)	140 (138; 141.3)	0.03
Diastolic pressure (mmHg)	65 (60; 75)	70 (60; 80)	0.02

Procedure-related conditions			
Time of cardiopulmonary bypass (min)	80 (62; 111)	77 (59; 100)	0.01
Lowest pO_2 _(mmHg)	105.0 (79; 252)	83.8 (73.5; 97)	<0.001
Lowest Na^+^ (mmol/L)	136 (134; 138)	137 (135; 139)	<0.001
Lowest K^+^ (mmol/L)	3.78 (3.6; 4)	3.7 (3.5; 3.9)	<0.001
ΔpH	0.05 (0.018; 0.09)	0.042 (0.007; 0.09)	0.01
ΔpO_2_ (mmHg)	19.2 (0; 164.7)	0 (0; 25.2)	<0.001
ΔNa (mmol/L)	3.1 (2; 5)	3 (1; 4)	<0.001
ΔK (mmol/L)	0.15 (0.04; 0.3)	0.1 (0; 0.24)	<0.001
ΔHematocrit (%)	14 (12; 17)	13 (10; 16)	0.09

pO_2_: arterial oxygen partial pressure, Δ: intraoperative fluctuation (max − min).

**Table 5:** Independent predictors of delirium in a multivariate analysis.

Variable	Logistic OR	95% CI	“*P*”
Postoperative cerebral ischemia (yes = 1)	2.862	1.391–5.890	0.004
Any blood transfusion (yes = 1)	4.178	2.422–7.207	<0.0001
Age > 65 years (yes = 1)	2.417	1.365–4.280	0.002
Carotid artery stenosis (yes = 1)	2.145	1.118–3.878	0.01
Urgent and emergent mode of surgery (yes = 1)	1.982	1.098–3.578	0.02
Hypertension (yes = 1)	0.406	0.231–0.714	0.002
Fasting glucose level (per 1 mg/dL)	1.006	1.002–1.011	0.006
ΔpO_2_ (<ΔQ1 or >ΔQ3 versus ΔQ1–3)	1.010	1.007–1.013	<0.001
ΔHematocrit (<ΔQ1 or >ΔQ3 versus ΔQ1–3)	1.065	1.005–1.129	0.03

OR: odds ratio, CI: confidence interval, pO_2_: arterial oxygen partial pressure, Δ: intraoperative fluctuation (max − min).

**Table 6:** Independent predictors of delirium in a multivariate analysis-subanalysis in patients who underwent CABG and/or valve procedure.

Variable	Logistic OR	95% CI	“*P*”
Obstructive pulmonary disease (yes = 1)	4.809	1.264–18.296	0.02
Any blood transfusion (yes = 1)	3.904	2.054–7.420	<0.001
Postoperative cerebral ischemia (yes = 1)	2.743	1.211–6.210	0.01
Carotid artery stenosis (yes = 1)	2.491	1.272–4.878	0.008
Age > 65 years (yes = 1)	2.307	1.162–4.580	0.01
ΔpO_2_ (<ΔQ1 or >ΔQ3 versus ΔQ1−3)	1.011	1.008–1.015	<0.001
Liver disease in anamnesis (yes = 1)	0.104	0.011–0.964	0.046

OR: odds ratio, CI: confidence interval, pO_2_: arterial oxygen partial pressure, Δ: intraoperative fluctuation (max − min).
